# Allergic diseases of the skin and drug allergies – 2018. Risk factors for hypoproteinemia in infantile atopic dermatitis

**DOI:** 10.1186/1939-4551-6-S1-P105

**Published:** 2013-04-23

**Authors:** Geunhwa Park

**Affiliations:** 1 Busan St. Mary's Medical Center, South Korea

## Background

Hypoproteinemia is one of complications of severe atopic dermatitis. The aim of this study was to investigate the risk factors for hypoproteinemia in atopic dermatitis.

## Methods

Seventy seven infants with atopic dermatitis were enrolled, who visited pediatric allergy clinic of Busan St. Mary’s Medical Center from January 2005 to January 2012. Infants with serum protein level was lower than normal range for age were classified to Group A (n=27) and normalto Group B (n=50). Age, sex, family history of allergy, and SCORAD score were studied. We examined platelet count, CRP, eosinophil count, serum albumin and protein, also serum ECP, total IgE, and allergen specific IgE by immuno CAP system (Phadia AB, Uppsala, Sweden). We identified skin wound culture and mycoplasma infection.

## Results

Group A (Atopic dermatitis with hypoproteinemia) had higher SCORAD score, eosinophil count, CRP, ECP, and total IgE, and lower albumin than control group. Serum protein had statistically significant correlations with SCORAD score, eosinophil count, albumin, total IgE and number of sensitized allergens, but had not with CRP and ECP.

## Conclusions

The lower serum protein, the more severe atopic dermatitis. Our study suggests that the risk factors for hypoproteinemia in infantile atopic dermatitis were high SCORAD score, eosinophil count, total IgE, and number of sensitized allergens, and low albumin.

